# N6-Methyladenosine (m^6^A)-Related lncRNAs Are Potential Signatures for Predicting Prognosis and Immune Response in Lung Squamous Cell Carcinoma

**DOI:** 10.1155/2022/5240611

**Published:** 2022-09-02

**Authors:** Yang Zhou, Xuhui Guan, Shuncong Wang, Huanhuan Sun, Haiqing Ma

**Affiliations:** ^1^School of Medicine, South China University of Technology, Guangzhou 510006, China; ^2^Guangdong Provincial People's Hospital, Guangdong Academy of Medical Sciences, Guangzhou, China; ^3^KU Leuven, Biomedical Group, Campus Gasthuisberg, Leuven 3000, Belgium

## Abstract

**Background:**

Despite increasing understanding of m^6^A-related lncRNAs in lung cancer, the role of m^6^A-related lncRNAs in the prognosis and treatment of lung squamous cell carcinoma is poorly understood to date. Thus, the current study aims to elucidate its role and build a model to predict the prognosis of LUSC patients.

**Materials and Methods:**

The data of the current study were accessed from the TCGA database. Pearson correlation analysis was performed to identify lncRNAs correlated to m^6^A. Next, an m^6^A-related lncRNAs risk model was built using a single factor, least absolute association, selection operator, and multivariate Cox regression analysis.

**Results:**

The relevance between 23 m^6^A genes and 14,056 lncRNAs is shown by Pearson correlation analysis by Sankey diagram. Multivariate Cox regression analysis determined that 11 m^6^A-lncRNAs show predictive potential in prognosis, which is confirmed by the consistency index, Kaplan–Meier analysis, principal component analysis, and ROC curve. Additionally, the immune analysis showed that the enrichment of immune cells, major histocompatibility complex molecules, and immune checkpoints in the high and low-risk subgroups were markedly disparate, with the high-risk group showing a stronger immune escape ability and a worse response to immunotherapy.

**Conclusion:**

In conclusion, the risk model based on m^6^A-related lncRNAs showed great promise in predicting the prognosis and the efficacy of immunotherapy.

## 1. Introduction

Lung cancer has long been the most fatal and the second most common malignancy globally [1]. LUSC accounts for 35% of nonsmall cell lung cancer (NSCLC) cases and shows unique epidemiological, clinicopathological, and molecular characteristics. For instance, it is closely related to smoking, low EGFR mutation rate, and low ALK rearrangement rate, leading to a poor targeted therapy outcome [2]. However, in recent years, as tumor immunotherapy strategies continue to improve, it has been reported that immunotherapy could be effective in LUSC, regardless of the PD-L1 expression and TMB levels [3–7]. At present, with the advent of more and more antitumor drugs, methods to improve the effect of antitumor drugs, especially immunotherapy, have also gratifying results, such as using nanotechnology as a carrier [8–12]. Therefore, identifying biomarkers that could accurately predict patient prognosis and efficacy of immunotherapy is urgently needed.

In eukaryotic cells, N6-methyladenosine (m^6^A), which participates in RNA biogenesis and function, is the most abundant RNA modification. Importantly, it mediates the modification of noncoding RNA (ncRNA) through various biological components [13]. At the same time, noncoding RNAs can reversely affect tumor progression and metastasis by regulating the m^6^A modification of mRNAs [13]. For example, FOXM1-ASlncRNA transcribed from the antisense strand of the FOXM1 gene can promote the process of tumorigenesis of GSCs by promoting ALKBH5 (an m^6^A erasure element) to remove m^6^A [14]. In addition, lncRNA targeting the reduction of GATA3 expression by inducing pre-mRNAKIAA1429-mediated m^6^A modification is needed for liver cancer cell development [15]. LncRNA Gas5-AS1 interacting with Gas5 promotes the ALKBH5-mediated m^6^A demethylation process, resulting in the expression of the tumor suppressor GAS5 and thus impeding the division and invasion of cervical cancer cells [16]. Taken together, m^6^A and lncRNA are highly correlated and might affect tumor growth and metastasis through their interaction. Additionally, m^6^A modification is a kind of epigenetic behavior tightly related to lung cancer and the m^6^A regulatory factor gene has a significant value in predicting prognosis for LUSC [17, 18]. Specifically, lncRNA-ATB might influence LUSC progression by controlling the microRNA-590-5p/NF-90 axis [19]. Similarly, some lncRNAs are underlying factors for predicting the prognosis in LUSC [20]. Nevertheless, the role of m^6^A-lncRNA in LUSC remains elusive.

Therefore, our study aims to explore whether m^6^A-lncRNAs could play an important predictive role in LUSC and to find potential markers of immunotherapy through immune-related analysis for screening large patient populations. In addition, we have also identified candidate drugs related to immunotherapy with significant differences in IC50 under this model.

## 2. Materials and Methods

### 2.1. Transcriptome and Clinical Data Acquisition

VarScan software was used to obtain the clinical information (gender, age, TNM stage, survival status, and survival time) and gene expression profile data of 505 patients with LUSC from the TCGA database.

### 2.2. Screening of m^6^A-Related lncRNAs

The expression matrix of 23 m^6^A-related genes was screened from the TCGA database [21]. Pearson pertinence analysis was applied to identify lncRNAs of interest, and a total of 2350 lncRNAs related to m^6^A were subsequently selected (|Pearson R| > 0.4 and *p* < 0.001).

### 2.3. Establishment of the Risk Model

The entire clinical dataset extracted from TCGA was stochastically split into two groups (training subgroup and testing subgroup). The baseline characteristics (gender, age, stage, and TNM stage) of the two subgroups showed no significant differences (*p* > 0.05). A risk model was then constructed using the training subgroup and verified using the testing and entire subgroups.

To classify the risk level, we used 11 m^6^A-related lncRNAs that adequately made contact with OS to score the risk of patients of the training set. Screening of m^6^A-related lncRNAs involved univariate Cox regression analysis as well as LASSO-penalized Cox analysis (using R language package GLMNET) and multivariate Cox ratio hazard regression analysis [22–24]. Risk score = Expr (lncRNA1) × Coef (lncRNA1) + ...... + Expr (lncRNAn) × Coef (lncRNAn) [23].

### 2.4. Accuracy Testing of This Model in Predicting Prognosis

We test the accuracy of the model by drawing C-index and receiver operating characteristic curve with the *R* package “timeROC.” [22] 1, 3, and 5 years OS was predicted with the scores acquired by scoring factors that affected prognosis (age, gender, stage, TNM stage, and risk score). We used the R language package “regplot” to draw the alignment diagram [25].

### 2.5. Independence Test of the Risk Model

To examine whether risk scores could be used as prognosis predictors like other clinical characteristics, univariate Cox and multivariate Cox analyses of the entire set of samples were performed [26].

### 2.6. PCA Analysis

PCA analysis was performed on the entire gene expression profile, 23 m^6^A-related genes, 2350 m^6^A-related lncRNAs, and the risk model to identify the sample difference and reduce high-dimensional data. The R language packages “scatterplot3d” and “limma” were used, respectively [27].

### 2.7. Immune Function Analysis

First, the gene expression discrepancy between the high-risk group and the low-risk group in the entire dataset with the help of the R language package “limma” was analyzed. Next, the clustering condition of genes that expressed discrepantly was observed by conducting GO analysis using the R package “clusterProfiler” to detect enrichment in different biological processes. [21] The critical value was 0.05. A *p* value less than the threshold revealed which GO terms were markedly clustered [26, 28].

### 2.8. Immunotherapy and Potential Drug Screening Analysis

The tumor immune dysfunction and rejection score (TIDE) is a calculation framework designed by Peng Jianget al. to integrate different tumor escape mechanisms. The effective samples and TIDE scores were obtained from https://tide.dfci.harvard.edu/ [22]. To explore the potential of therapeutic drugs, the IC50 of the compound obtained from the GDSC website in LUSC patients was predicted using the R language package “pRophetic” [21].

## 3. Results

### 3.1. Extracting m^6^A-Related lncRNA from LUSC Patients

**Figure 1:**
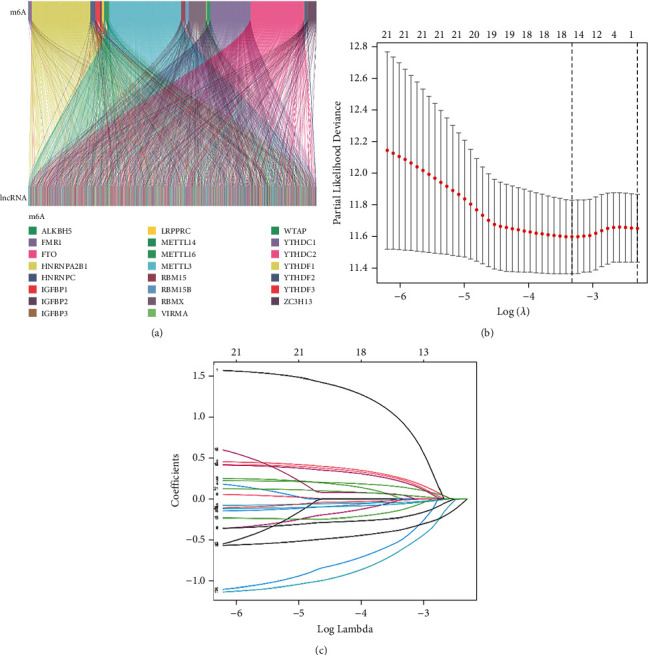
Construction of the risk model based on m6A-related lncRNAs for LUSC patients. (a) Sankey diagrams of 23 m^6^A genes and m^6^A-related lncRNAs. (b)-(c) Least absolute shrinkage and selection operator (LASSO) analysis of 16 m^6^A-related lncRNAs that affected prognostic. Establishment of the m6A-related lncRNAs risk model.

We extracted 23 m^6^A genes and 14056 lncRNAs. LncRNAs significantly related to one or more 23 m^6^A genes were termed m^6^A-related lncRNAs. 2350 lncRNAs related to m^6^A were obtained, and a Sankey diagram was drawn to observe the potential association between m^6^A genes and lncRNAs (Figure 1(a)).

First, univariate Cox regression analysis was exerted to screen m^6^A-related lncRNAs that have a significant correlation with overall survival (Figure S1). Lasso-Cox regression analysis was then applied to accurately and effectively identify predictive markers based on the LASSO-penalized regression model to identify lncRNAs related to overall survival according to the smallest lambda value. The selected m^6^A-related lncRNAs (*n* = 16) were incorporated into multivariate regression analysis (Figures 1(b) and 1(c)).

**Figure 2:**
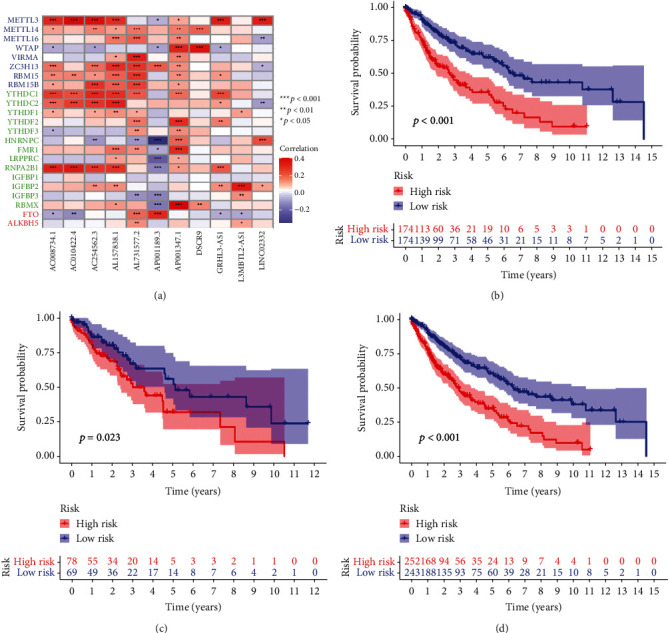
Correlational heatmap and Kaplan–Meier survival analysis. (a) Heatmap for the correlations between 23 m^6^A genes and the 11 prognostic m^6^A-related lncRNAs. (b) KM survival curve of the model in the training set, (c) testing set, and (d) entire set.

Finally, 11 m^6^A-lncRNAs independently related to OS were used to construct the risk models (Table 1).  Figure 2(a) illustrates the correlation between the m^6^A genes and lncRNAs used for model construction.

**Figure 3:**
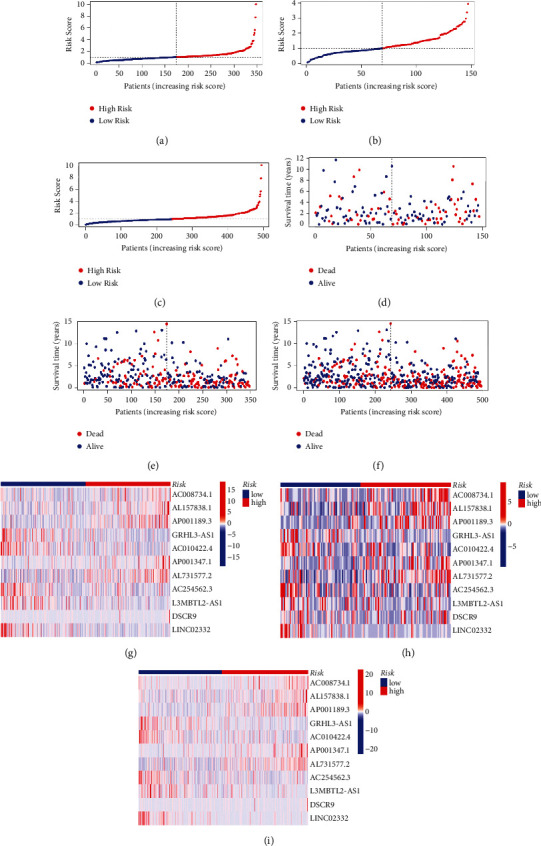
The relationship between the m6A-related lncRNAs risk model and prognosis is verified in the training set, testing set, and entire set. (a) Distribution of risk scores in the training set, (b) testing set, and (c) entire set. (d) Distribution of survival status in the training set, (e) testing set, and (f) entire set. (g) Heatmap of 11 m^6^A-lncRNAs in the testing set, (h) training set, and (i) entire set.

To assess the potential prognostic value of these m^6^A-related lncRNAs, 495 patients obtained from the TCGA database were stochastically separated into training and testing groups (Table 2). The training group was used to establish the model and predict its accuracy, while the validation group and the whole dataset were used to verify the model. Based on the median value of the risk score of each group, the LUSC samples were split into high and low-risk subgroups, and K-M survival curves were drawn (Figures 2(b)–2(d)). Next, we analyzed the distribution of risk levels in each group and displayed the status of patients in each subgroup via a dot chart. Finally, a heatmap was generated to visualize the expression patterns of the 11 lncRNAs in two subgroups (Figures 3(a)–3(i)).

The results of the training set analysis suggested that patients in the high-risk group had lower overall survival rates than the low-risk group (*p* < 0.01). Similar results were obtained when the validation and whole datasets were analyzed. Accordingly, our model has good potential for prognosis prediction.

### 3.2. Test the Accuracy of the Risk Model in Predicting the Prognosis and Classification

**Figure 4:**
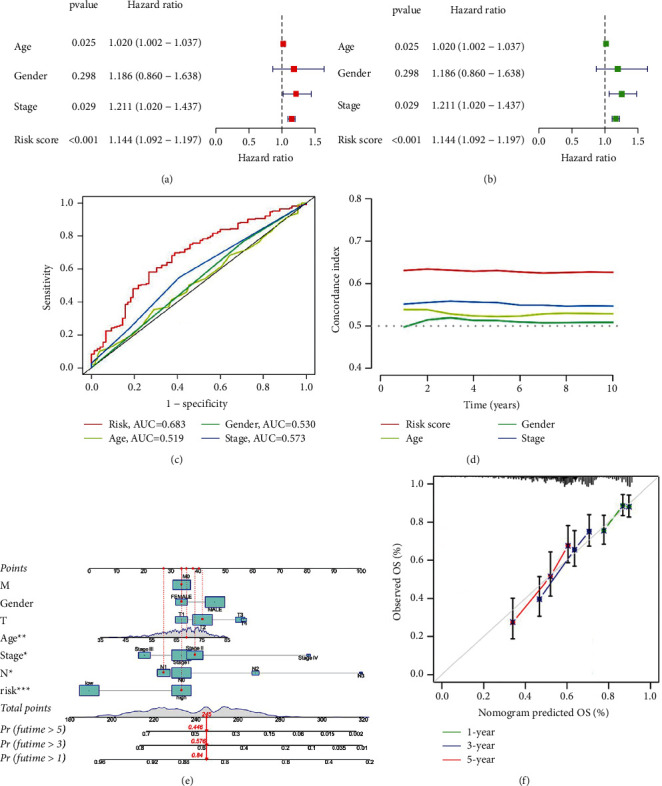
Evaluation of the risk model of the m6A-related lncRNAs. (a) Multivariate prognostic analysis of risk score and clinical characteristic. (b) Univariate prognostic analysis of risk score and clinical characteristic. (c) ROC curves of risk score and clinical characteristics. (d) C-index of risk score and clinical characteristic. (e)-(f) Nomogram and calibration curve predicted the overall survival of 1-year, 3-year, and 5-year grounded on gender, age, TNM, and risk score (Example: female, 69 years old, stage II, T2M0N1, high risk). Application of risk model for tumor immune-related analysis.

The results from the whole dataset further underwent univariate and multivariate Cox regression analyses, revealing that the risk score is an independent prognostic factor (Figures 4(a) and 4(b)). To accurately illustrate the universality and importance of the risk model in forecasting prognosis, the concordance index (C-index) of risk scores and AUC were evaluated. It was found that the AUC of the risk score was higher compared to clinical characteristics such as age, gender, and tumor stage, with the C-index further indicating good consistency between predicted and actual observations (Figures 4(c) and 4(d)). Nomograms and calibration curves based on age, gender, TNM, and risk score were also drawn to predict the OS of patients at 1, 3, and 5 years, which validated the high accuracy and authenticity of our model (Figures 4(e) and 4(f)).

**Figure 5:**
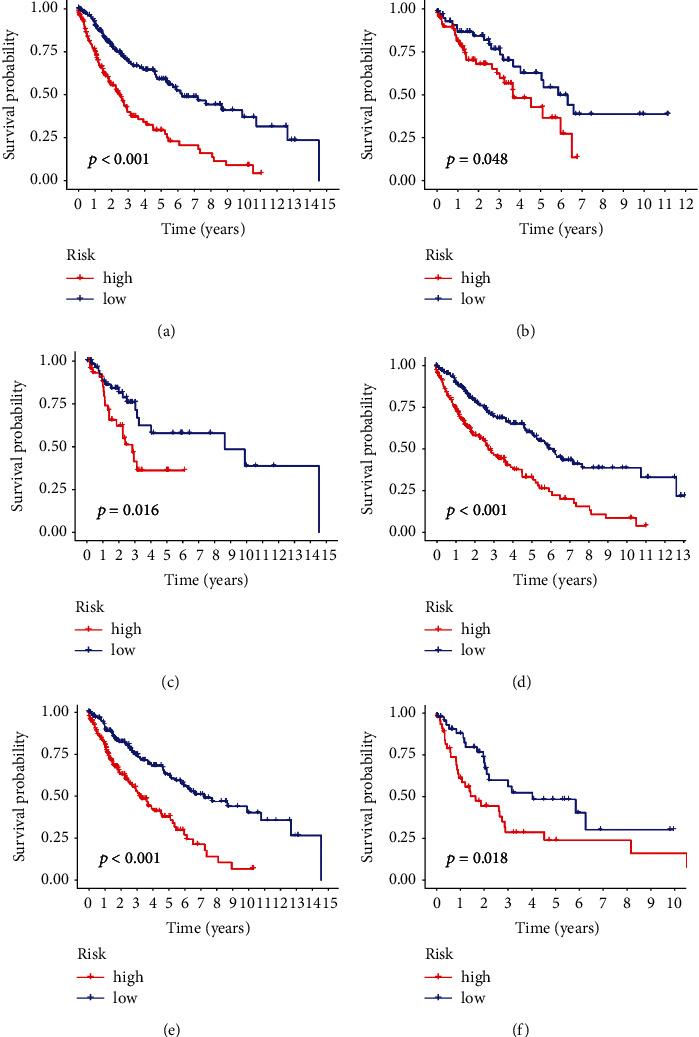
Survival analysis based on gender, age, stage, and other multiple clinicopathological characteristics between the high-risk and low-risk groups in the entire set. Evaluation of immunotherapy effects and drug screening based on models.

To assess whether our model is appropriate for patients with different clinicopathological characteristics, the difference between high and low-risk subgroups of OS was analyzed by stratifying according to different clinicopathological characteristics. The results that applying K-M analysis to analyze the entire group of samples based on three clinicopathological characteristics of age, gender, and stage demonstrated that OS in high-risk group was lower, compared to the low-risk subgroup (Figures 5(a)–5(f)). Moreover, similar results were observed after stratifying by TNM staging and tumor mutation burden (Figures S2A and 5(h)).

**Figure 6:**
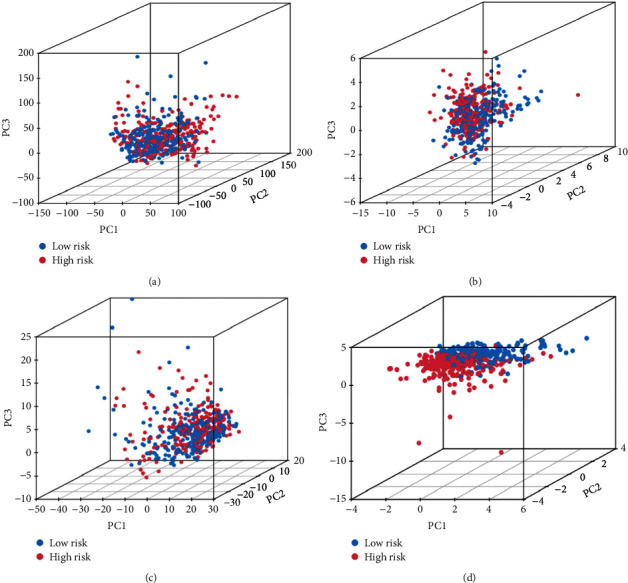
PCA analysis in a high-risk group and low-risk group grounded on (a) the whole gene expression profile. (b) 23 m^6^A-related genes. (c) 2350 m^6^A-related lncRNAs. (d) 11 m^6^A-related lncRNAs.

PCA analysis was carried out on the whole gene expression profile, 23 m^6^A-related genes, 2350 m^6^A-related lncRNAs, and 11 m^6^A-related lncRNAs to verify that our model was superior to other models and to assess its ability to distinguish patients with different risk levels. It was found that the degree of distinction between the two subgroups was higher in our model compared with the other three models, which enabled better differentiation between high and low-risk subgroups (Figures 6(a)–6(d)).

**Figure 7:**
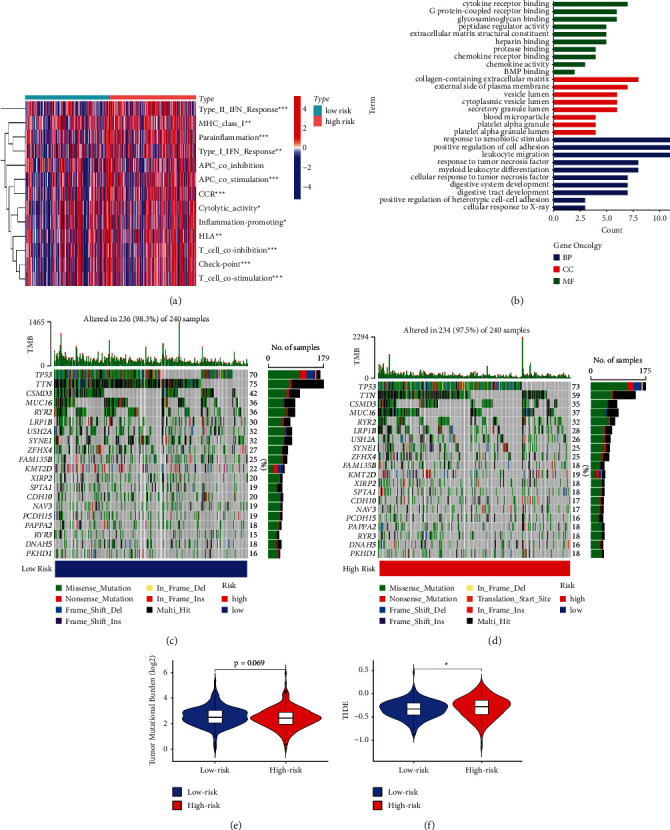
Exploring molecular mechanism and prediction of immunotherapy response in the entire set. (a) Enrichment level of immune cells, immune pathways, and immune function in the high- and low-subgroups. (b) Gene ontology (GO) enrichment analysis. The top 20 genes with mutation frequency between (c) the high-risk and (d) low-risk groups in the entire set. (e) TMB discrepancy between high-risk and low-risk groups. (f) TIDE scores discrepancy between high-risk and low-risk groups.

98 differential genes were screened by comparing the differences of genes between the high and low-risk subgroups in the whole dataset to identify the potential molecular mechanism of the m^6^A-based model. GO enrichment analysis indicated the biological processes were enriched in immunity (Figure 7(a)). Next, the immune enrichment results of immune cells, immune pathways, major histocompatibility complex molecules, chemokine receptors, and immune checkpoints indicated that the immune system of the high-risk subgroup was more active (Figure 7(b)). 20 driver genes with the most frequent alteration between the two subgroups were identified (Figures 7(c) and 7(d)). Furthermore, the TMB scores calculated from the TGCA dataset showed no significant differences (*p*=0.069) (Figure 7(e)).

The TIDE scores of all cases are based on the expression levels of immunotherapy biomarkers such as IFNG, MSI, Merck18, CD274, CD8, CTL, MDSC, CAF, and TAM-M20, suggesting that immune escape function in the high-risk group is stronger and a worse response to immunotherapy. This finding suggests that our model can classify patients by predicting their response to immunotherapy (Figure 7(f)).

Finally, 10 out of 78 compounds, with the most significant difference in drug half-maximal inhibitory concentration between high and low-risk groups, were screened to identify possible therapeutic drugs with our model (Figures S3A and 7(j)), which provides the basis for follow-up studies on therapeutic drugs for LUSC [29].

## 4. Discussion

Poor understanding of driver genes in LUSC accounts for the limited number of treatment strategies for this patient population [30, 31]. Hence, accurate prediction of the prognosis of the patients with LUSC is necessary, emphasizing the need to identify biomarkers for guiding treatment. It has been shown that m^6^A modifications and lncRNAs influence the occurrence and development of LUSC [32, 33].

In the present study, a risk model that works on predicting the prognosis in LUSC was established, and the relationship between our model and immune response was explored. An increasing body of evidence suggests that m^6^A-related lncRNAs are tightly related to antitumor immunity and immune infiltration [34, 35].

11 out of 2350 m^6^A-related lncRNAs that correlated with OS were screened. Among these, AP001189.3 has been reported to be related to MAPK and other signaling pathways and could reportedly predict the prognosis of colon cancer [36]. L3MBTL2 promotes the recruitment of the ubiquitin ligase RNF168 to DNA lesions and promotes the repair process. Meanwhile, L3MBTL2 can also serve as a key target of the ubiquitin ligase RNF8 after DNA damage [37]. However, L3MBTL2 has rarely been reported as a prognostic factor. Our research can provide a new direction for future generations to further study the function of this gene. Furthermore, GRHL3-AS1 was reported to have a prognostic function in primary head and neck squamous cell carcinoma [38]. Nonetheless, to the best of our knowledge, the predictive effect and biological function of the remaining 8 lncRNAs (AC008734.1, AL157838.1, AC010422.4, AP001347.1, AL731577.2, AC254562.3, DSCR9, and LINC02332) have not been reported in the literature. Indeed, the present study results provide the basis for future studies on the molecular biological function of these m6A-related lncRNAs in the occurrence and progression of LUSC.

The 495 cases extracted from TCGA were split into two groups based on the median risk score. Importantly, we found that the OS was lower in the high-risk group than in the low-risk one. Additionally, when stratified by gender, stage, TNM stage, and TMB, the OS in the high-risk group was still poorer, compared to the low-risk group. Therefore, our risk model consisting of 11 m^6^A-related lncRNAs correlated with OS yielded accurate results and provided the basis for subsequent research on potential biomarkers for LUSC treatment. Moreover, the analysis of GO enrichment indicated that the immune system in the high-risk group was more active, suggesting the potential relationship between our model and immune response. Moreover, this finding substantiated the association between the poor clinical outcome of the high-risk subgroup and the induction of highly expressed immune molecules. The TIDE score suggested that the high-risk subgroup reacted worse to immunotherapy because of a stronger immune escape function, compared to the low-risk subgroup, which could be attributed to a stronger immune escape ability. It is noteworthy that the expression of immune-related molecules in the low-risk subgroup was relatively lower. The above results indicate that immunotherapy can be effective, even in patients that express fewer immune checkpoint molecules such as PD-L1, compared with those with high expression of PD-L1, consistent with the literature [11, 31, 39]. Our results can also explain the phenomenon whereby some PD-L1 < 1% patients exhibit a better response to immunotherapy in contrast to PD-L1 > 1% patients [12]. The present study results are expected to offer novel insights into the biological function of m^6^A-related lncRNAs in LUSC. It has been shown that titin (TTN) is expressed in both groups, except TP53. Previous studies have shown that TTN can be alone considered a factor predicting the prognosis of LUSC and the efficacy of the treatment with immune checkpoint inhibitors.

Herein, univariate, LASSO, and multivariate Cox regression analyses were initially made use of selecting m6A-related lncRNAs with predictive function. Moreover, C-index, AUC, and KM analysis confirmed the powerful prediction ability of the risk model to predict prognosis.

However, this study also has certain limitations. Indeed, our model was established after multiple screenings, and the sample size was limited; more external experimental verification is needed to substantiate that m^6^A-related lncRNAs are efficient predictive biomarkers. What is more, the interaction between these prognostic lncRNAs and m^6^A regulatory factors in LUSC was not explored, and it remains unclear how m^6^A-lncRNAs can affect tumor immune response, warranting the need for further studies.

In conclusion, we established a model playing a prognostic role, made of 11 m^6^A-related lncRNAs from TCGA related to the tumor immune response, providing new directions for the prediction of patient prognosis in LUSC. Importantly, our model may help screen patients with good responses to immunotherapy and even clarify the biological processes of m^6^A-related lncRNAs in LUSC.

## Figures and Tables

**Table 1:** 11 m^6^A-related lncRNAs that established the risk model.

Id	Coefficient	Hazard ratio
AC008734.1	1.651695304	3.126981553
AL157838.1	0.428749482	1.358522989
AP001189.3	0.288440222	1.492333973
GRHL3-AS1	−0.443479624	0.524775133
AC010422.4	−1.063521258	0.287925081
AP001347.1	0.427439556	1.606430239
AL731577.2	0.500702103	1.734871772
AC254562.3	−0.392242562	0.695193798
L3MBTL2-AS1	−0.126364666	0.843723126
DSCR9	0.133435626	1.138092172
LINC02332	−0.695292467	0.387123601

**Table 2:** Characteristics of LUSC patients in training, testing, and entire sets from TCGA database.

Characteristics	Type	Total	Test	Train	*P* value
Age	≤65	189 (38.18%)	63 (42.86%)	126 (36.21%)	0.1632
	>65	300 (60.61%)	81 (55.1%)	219 (62.93%)	
	Unknown	6 (1.21%)	3 (2.04%)	3 (0.86%)	

Gender	Female	129 (26.06%)	40 (27.21%)	89 (25.57%)	0.7896
	Male	366 (73.94%)	107 (72.79%)	259 (74.43%)	

Stage	Stage I	242 (48.89%)	68 (46.26%)	174 (50%)	0.5604
	Stage II	159 (32.12%)	53 (36.05%)	106 (30.46%)	
	Stage III	83 (16.77%)	21 (14.29%)	62 (17.82%)	
	Stage IV	7 (1.41%)	2 (1.36%)	5 (1.44%)	
	Unknown	4 (0.81%)	3 (2.04%)	1 (0.29%)	

*T*	T1	114 (23.03%)	30 (20.41%)	84 (24.14%)	0.718
	T2	288 (58.18%)	91 (61.9%)	197 (56.61%)	
	T3	70 (14.14%)	19 (12.93%)	51 (14.66%)	
	T4	23 (4.65%)	7 (4.76%)	16 (4.6%)	

*M*	M0	407 (82.22%)	117 (79.59%)	290 (83.33%)	1
	M1	7 (1.41%)	2 (1.36%)	5 (1.44%)	
	Unknown	81 (16.36%)	28 (19.05%)	53 (15.23%)	

*N*	N0	316 (63.84%)	98 (66.67%)	218 (62.64%)	0.4745
	N1	128 (25.86%)	31 (21.09%)	97 (27.87%)	
	N2	40 (8.08%)	13 (8.84%)	27 (7.76%)	
	N3	5 (1.01%)	2 (1.36%)	3 (0.86%)	
	Unknown	6 (1.21%)	3 (2.04%)	3 (0.86%)	

## Data Availability

TCGA belongs to public databases. Users can download relevant data for free for research and publish relevant articles.

## Supplementary Materials

Supplementary Materials Figure S1: Univariate ratio risk Cox regression screened out 21 m6A-related lncRNAs. Figure S2: Kaplan–Meier survival analysis based on TNM and TMB, between the high-risk and low-risk groups in the entire set. Figure S3: 10 candidate compounds targeting the m6A-related lncRNA prognosis model.
