# Supplementary figures and images for: N6-Methyladenosine (m6A)-Related lncRNAs Are Potential Signatures for Predicting Prognosis and Immune Response in Lung Squamous Cell Carcinoma
Source: J Oncol. 2022 Sep 2;2022:5240611. doi: 10.1155/2022/5240611 (PMC9462982; doi:10.1155/2022/5240611)

Figure S1

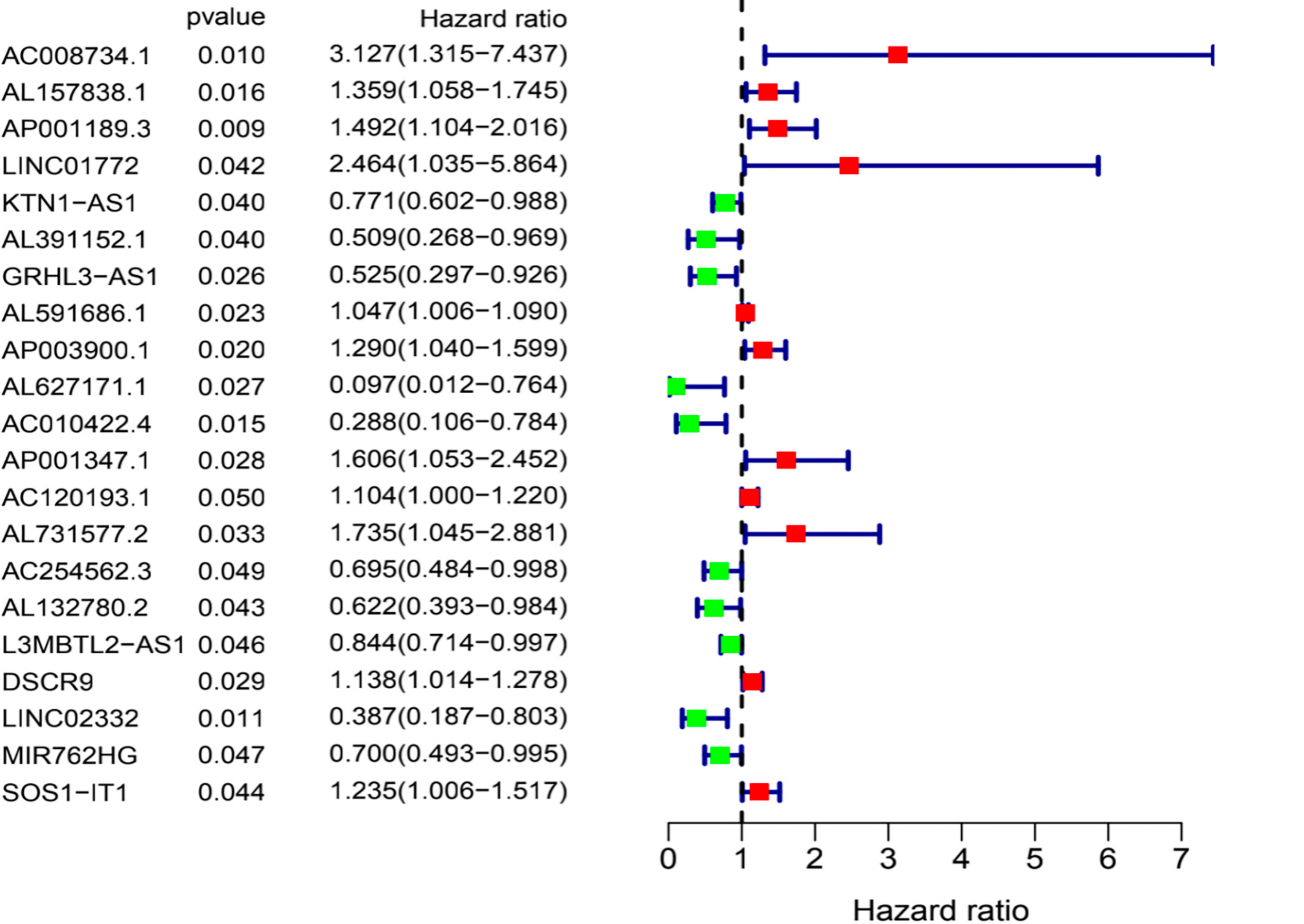

Supplement: Supplementary Materials — Figure S1: Univariate ratio risk Cox regression screened out 21 m6A-related lncRNAs. Figure S2: Kaplan–Meier survival analysis based on TNM and TMB, between the high-risk and low-risk groups in the entire set. Figure S3: 10 candidate compounds targeting the m6A-related lncRNA prognosis model. [file 5240611.f1.zip › Figure_S1.pdf]

Figure S2

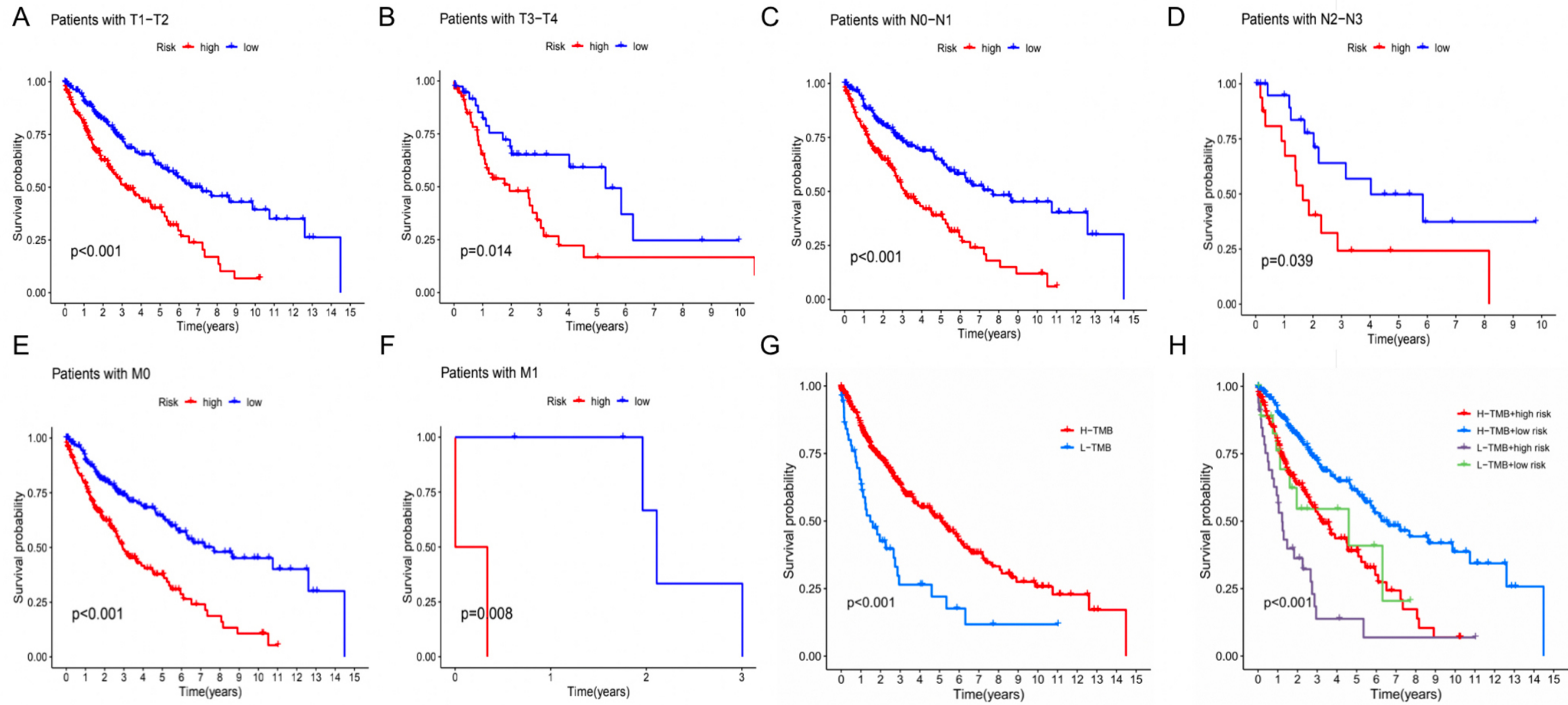

Supplement: Supplementary Materials — Figure S1: Univariate ratio risk Cox regression screened out 21 m6A-related lncRNAs. Figure S2: Kaplan–Meier survival analysis based on TNM and TMB, between the high-risk and low-risk groups in the entire set. Figure S3: 10 candidate compounds targeting the m6A-related lncRNA prognosis model. [file 5240611.f1.zip › Figure_S2.pdf]

**Figure S3**

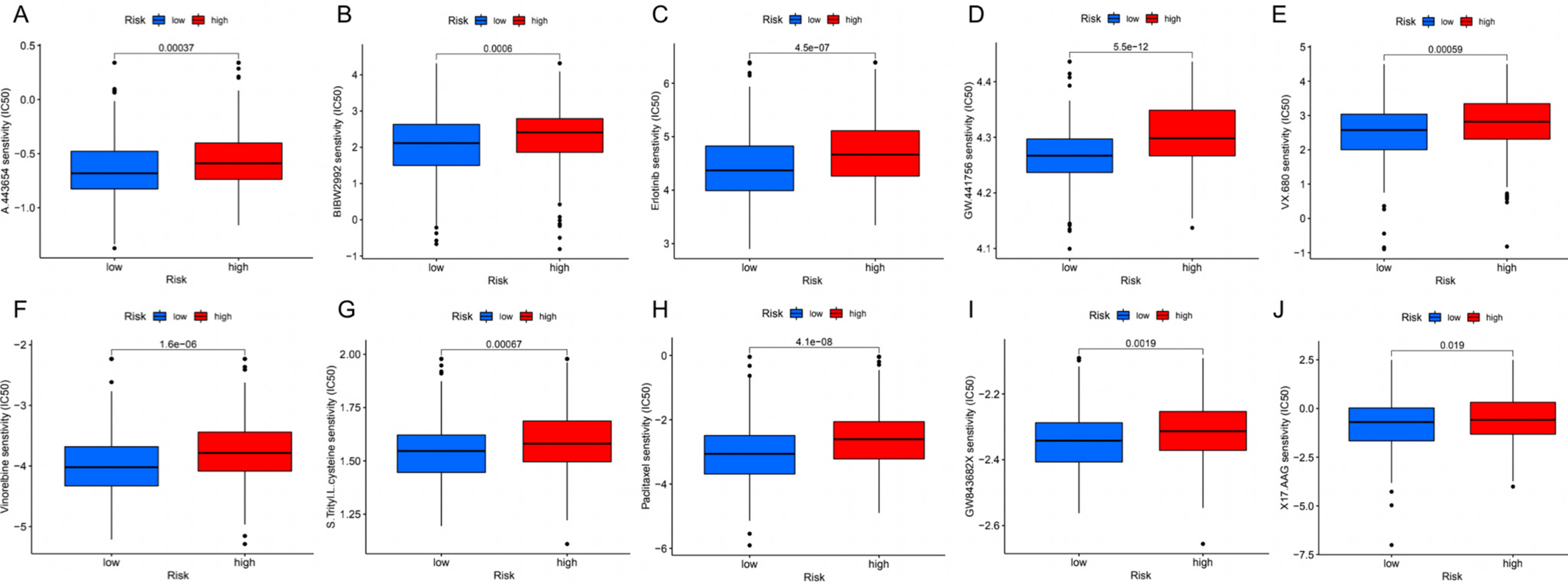

Supplement: Supplementary Materials — Figure S1: Univariate ratio risk Cox regression screened out 21 m6A-related lncRNAs. Figure S2: Kaplan–Meier survival analysis based on TNM and TMB, between the high-risk and low-risk groups in the entire set. Figure S3: 10 candidate compounds targeting the m6A-related lncRNA prognosis model. [file 5240611.f1.zip › Figure_S3.pdf]
